# Anticoagulation Stewardship to Bridge the Implementation Gap in Perioperative Anticoagulation Management

**DOI:** 10.1055/a-2259-0911

**Published:** 2024-03-11

**Authors:** Alfonso J. Tafur, Geoffrey D. Barnes, Vinai C. Bhagirath, James Douketis

**Affiliations:** 1 Department of Medicine, Vascular Medicine, NorthShore—Edward-Elmhurst Health, Evanston, Illinois, United States; 2 Department of Medicine-Cardiovascular Medicine, Pritzker School of Medicine, University of Chicago, Chicago, Illinois, United States; 3 Frankel Cardiovascular Center and Institute for Healthcare Policy and Innovation Department of Internal Medicine, University of Michigan Medical School, Ann Arbor, Michigan, United States; 4 Department of Medicine, McMaster University, Hamilton, Ontario, Canada

**Keywords:** perioperative, anticoagulation, implementation, six sigma, nudge, guidelines

## Abstract

Lack of alignment of care protocols among providers in health care is a driver of increased costs and suboptimal patient outcomes. Perioperative anticoagulation management is a good example of a complex area where protocol creation is a clinical challenge that demands input from multiple experts. Questions regarding the need for anticoagulation interruptions are frequent. Yet, due to layers of complexity involving analysis of anticoagulation indication, surgical risk, and anesthesia-associated bleeding risk as well as institutional practices, there is heterogeneity in how these interruptions are approached. The recent perioperative anticoagulation guidelines from the American College of Chest Physicians summarize extensive evidence for the management of anticoagulant and antiplatelet medications in patients who undergo elective interventions. However, implementation of these guidelines by individual clinicians is highly varied and often does not follow the best available clinical evidence. Against this background, anticoagulation stewardship units, which exist to improve safety and quality monitoring for the anticoagulated patient, are of growing interest. These units provide a bridge for the implementation of value-based, high-quality guidelines for patients who need perioperative anticoagulation interruption. We use a case to pragmatically illustrate the problem and tactics for change management and implementation science that may facilitate the adoption of perioperative anticoagulation guidelines.

A 74 kg (body mass index, 22), 65-year-old male is chronically anticoagulated with warfarin for an unprovoked proximal deep vein thrombosis that had occurred 3 years prior. He is heterozygous for factor V Leiden, has a creatinine clearance of 63 mL/min, and is scheduled for a laparoscopic hernia repair with mesh. The surgical team asked the patient to have a preoperative evaluation by his primary care physician (PCP), who is not affiliated with their institution. The PCP instructed him to stop warfarin 4 days before surgery, to use enoxaparin 80 mg BID starting 3 days before surgery and to use enoxaparin 40 mg BID (two times a day) starting the day after surgery along with warfarin until the international normalized ratio was 2 or higher.

The patient returned to the emergency department 5 days after surgery with extensive ecchymosis in his abdomen, penis, and scrotum.

## Introduction

For patients who are chronically anticoagulated for thrombotic conditions, including atrial fibrillation (AF) or venous thromboembolism (VTE), the perioperative period often requires temporary interruption of anticoagulants in a manner that minimizes the risks of both bleeding and thromboembolic complications. 1 2 3 4 These risks—which exist whether the patient is on a vitamin K antagonist, such as warfarin, or a direct oral anticoagulant (DOAC)—require consideration of pharmacodynamics, procedural risk assessment, type of anesthesia, and patient-specific thrombotic and bleeding risk. 5 Given the high prevalence of comorbidities associated with the growing age of chronically anticoagulated patients, approximately 15% of them need to go through an interruption process every year. 1 5 6 Moreover, risk factors for perioperative bleeding or thrombotic complications are also prevalent including cancer, anemia, thrombocytopenia, bleeding history, or extensive surgery. 1 7 8 Thus, even in carefully planned clinical trials focused on minimizing adverse events, major bleeding occurs in approximately 2 to 3% of patients, most often in the first 10 perioperative days. 4 9 10

To guide safe perioperative management of patients taking antithrombotic agents, the American College of Chest Physicians (ACCP) updated their Perioperative Antithrombotic Management guidelines in 2022 and considered 43 patient–intervention–comparator–outcome questions relating to perioperative anticoagulation care. 11 Unfortunately, there still remain gaps in guideline implementation in health care, 12 including in the application of guidelines for patients who need perioperative anticoagulation care. 13 Thus, between disciplines there is frequent heterogeneity on the degree of utilization of thrombotic and bleeding risk assessment, 13 14 utilization of longer than necessary anticoagulation interruption remains common, and use of bridging in scenarios where it is not recommended remains prevalent. 15 A similar implementation gap in the field of antimicrobial management led to the development of multidisciplinary teams known as antimicrobial stewardship programs. Over two decades of literature documents, the reduction of antibiotic-related drug events that followed the requirement by the Centers for Medicare and Medicaid Services to use antibiotic stewardship and inspired the structure of anticoagulation stewardship units. Aiming for improved safety and efficacy of anticoagulation management in a coordinated and sustainable way, anticoagulation stewardship units are tasked with leading the implementation of evidence-based anticoagulation care; orchestrating error-free administration of anticoagulants; and optimizing longitudinal monitoring of patients and their clinical response to anticoagulants. 16 In this review, we assess barriers to the implementation of perioperative anticoagulation guidelines, and how stewardship and implementation science concepts can improve the adoption of evidence-based perioperative anticoagulation guidelines.

## Cognitive Barriers to Implementation of Perioperative Anticoagulation Guidelines

Understanding our own cognitive biases can help explain why evidence alone does not modify action. There are two processes that coordinate action according to cognitive sciences as popularized by Kahneman in *Thinking Fast and Slow* . 17 Guideline creation and assessment is governed by type 2 thinking which is analytic, slow, and probabilistic. Decisions around an elective surgery often have a short lead-time and the care provider needs to decide on management during a brief phone call or message interaction rather than a paced medical appointment. The faster pace of many anticoagulation interruption decisions may be often a type 1 thinking process—more automatic, affect-based, fast, and narrative. 17 18 Consequently, clinicians often rely on heuristics developed during their training to facilitate a busy workflow and may not always incorporate the latest evidence or guideline recommendations.

The process of perioperative interruption also has a strong emotional bias anchored on prior experiences and availability heuristics. 19 The risk aversion is not uniform among providers. 14 Some specialists or even patients may attribute much higher value to stroke prevention, whereas the surgeon, PCP, and anesthesiologist may place a higher value on avoiding bleeding complications. The large number of specialties with interest in perioperative anticoagulation underscores the relevance of this topic, yet it also highlights the diversity of opinion on the topic. 20 21 22 23 24 25 This can lead to a lack of coordination between the preferences of multiple stakeholders and specialty groups who may provide conflicting advice to patients and health care staff. 21 Often, these groups may not realize that there is a downstream problem until data are presented to justify an urgent need for change. Thus, a multidisciplinary coalition to establish common ground and a shared vision of the problem is a key aspect of the perioperative anticoagulation interruption process. 26 The common denominator to an anticoagulation approach is (1) to decide if the procedure warrants anticoagulation or antiplatelet interruption; (2) if an interruption is needed, to estimate the case-specific major bleeding and thromboembolic risk that will inform anticoagulant interruption and resumption; and (3) among warfarin-treated patients, to determine the need for heparin bridging. 1 27 An anticoagulation stewardship unit could use this approach to forge institutional agreements across provider groups on perioperative antithrombotic management.

Creating institutional agreements on which minimal-risk interventions do not need interruption can reduce the downstream use of resources and eliminate unnecessary interruptions and overuse of anticoagulation bridging. Decisions in medicine always have a level of uncertainty; every decision carries the risk of error. Decision errors can be errors of action (commissions) or errors of inaction (omissions). In perioperative anticoagulation management, commission errors can lead to overuse, for example, compulsory bridging. However, assuming anticoagulation interruption is necessary for a procedure, the expert interpretation of current data by the ACCP suggested against a default bridging approach for most anticoagulation indications. 11 This stresses the need for awareness that physicians may have a bias toward commission, which in this case, further limits the implementation of evidence-based practice. In addition, although economically unsustainable, health care resource overuse is much more commonly tolerated than judicious avoidance of health interventions. 18 28

In the case vignette, the insistence on high dose bridging is an example of commission error. The perioperative risk attributed to factor V Leiden carriers on chronic anticoagulation is not an indication to provide aggressive bridging. 29 Moreover, if bridging is recommended, the last dose should not be on the evening before the procedure, as there will be residual effects on the day of surgery.

The perioperative anticoagulation guidelines distill scenarios in which interruptions or bridging do not add patient benefit. Yet, commission errors remain common. Anticoagulation stewardship units can systematically examine barriers to implementing evidence-based perioperative anticoagulation care.

## Anticoagulation Stewardship as a Tool for Care Pathway Alignment

Methods to facilitate the adoption of research findings into routine health care are studied in implementation science. Some of these tools (e.g., change management, health systems engineering principles, or nudges) have been used in anticoagulation stewardship and may aid in guideline alignment for perioperative anticoagulation. We provide a description of several different approaches to clinical care improvement along with relevant examples from the literature.

### Environmental Analysis

It is easy to miss stakeholders or potential barriers to implementation when we start problem-solving without using a systematic method to define it. An environmental analysis is a strategic technique to identify internal factors specific to the main team in the process, as well as external factors which are often outside the institution but may still affect the outcome. There are multiple tools to this effect; thus, a SWOT (Strengths, Weaknesses, Opportunities, and Threats) analysis will examine strengths and weaknesses as internal factors and opportunities and threats as external. The TOWS matrix elevates the SWOT tool to stimulate thinking about how to combine strengths to improve opportunities (maxi–maxi strategy) or to decrease threats (maxi–mini strategy). Similarly, it triggers the user to reconsider how to minimize weaknesses by taking advantage of opportunities (mini–max strategy) and avoiding threats (mini–min strategy).

In Table 1 , we present a Political, Economic, Sociocultural, Technological, Legal, and Environmental analysis to illustrate a structured method to examine barriers specific to perioperative anticoagulation care. 30

**Table 1:** PESTEL analysis in perioperative anticoagulation management

PESTEL	Examples	Implementation considerations
Political	Biases toward the need for periprocedural interruption of anticoagulation by different procedural teams. Areas of discrepancy between multiple existing guidelines. Barriers to interinstitutional agreement for centralization of care versus consulting primary prescriber.	Institutionally sponsored anticoagulation stewardship unit to consolidate and maintain agreement between multiple specialties on evidence-based indications for perioperative bridging. Share internal policies with referring centers and invite to discuss areas of concern.
Economic	Cost of parenteral anticoagulation. Selection of a full time equivalent dedicated to delivering perioperative anticoagulation instructions.	The anticoagulation stewardship can evaluate value-centered initiatives and report the time-driven activity-based costing (TDABC) analysis to maintain accountability in the comparative cost of the initiatives.
Social	Anticoagulation use is shared by multiple specialties. There is an emotional bias to perception of bleeding and thrombotic risk. Patients are often put in charge of asking the prescriber for instructions.	Facilitate evidence-based, internally approved order sets for facilitation and communication. Review institutional process and integrate lean thinking tactics to limit redundancy.
Technological	Limited integration of EMR for data absorption specific to procedure and to patient. The acceptance and efficacy of telehealth appointments in perioperative anticoagulation instructions is growingly used but not fully studied.	Leadership sponsoring and access to information technology collaboration are needed. While the pursuit of high quality and cost saving may justify the initial investment, ultimately value-based incentives are negotiated to sustain the unit.
Environmental	Procedures are often done in locations with protocols different to the anticoagulation prescriber. The speed to access to prescription medications can vary depending on patient location and insurance.	Evaluate social determinants of health as outcome modifiers for anticoagulation outcomes. Collaborate with the schedulers to optimize the lead time between procedure listing and delivery of anticoagulation instructions.
Legal	Compliance with health care regulations and standards is essential in anticoagulation management to ensure patient safety and avoid legal issues.	Early partnership with risk management when large initiatives are anticipated.

Abbreviations: EMR, electronic medical record; TDABC, time-driven, activity-based costing.

### Change Management

Successful quality improvement projects demand an understanding of change management principles. One of the most widely reported change management systems in health care is the Kotter model. 31 This eight-step model is anchored on creating a sense of urgency, building a powerful guide coalition, developing a strategic vision, communicating the vision, empowering action, creating short-term wins, integrating improvements, adjusting for more changes, and making change stick. 32

**Fig. 1:**
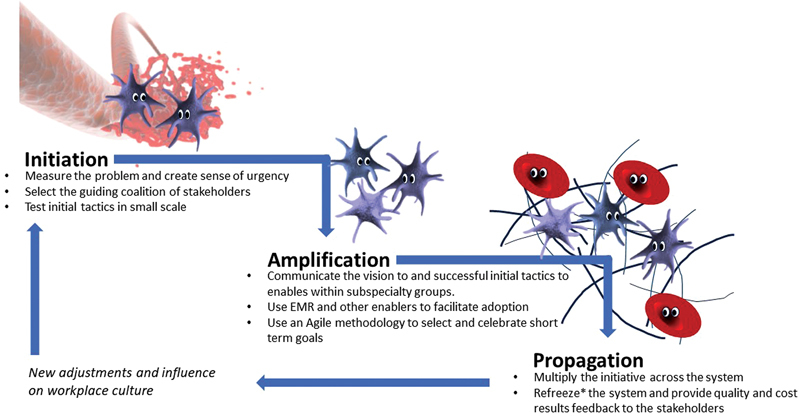
Change management from the anticoagulation stewardship perspective. Adapted from the Kotter model. In reference to cellular coagulation model. *Reference to Unfreeze, Change, Refreeze change management model by K Lewin.

Anticoagulation stewardship units trigger the initiation of the change management process by measuring the problem and evaluating potential solutions. After potential solutions are identified, we suggest that the anticoagulation stewardship unit works with stakeholders to select the appropriate implementation strategies to curate and then amplify the effort. These tactics may often need to be specific to the problem and to the culture of the institution. Finally, the unit identifies champions among the clinical services and facilitates system-wide decision support tools that can help propagate the new care pathways. Besides the initiation, amplification, and propagation process ( Fig. 1 ), the unit needs to create continued surveillance and reinforcement procedures to maintain or adapt the process.

In one example from a quality improvement initiative in the U.S. state of Michigan, the use of aspirin among patients with chronic anticoagulation with warfarin was audited, which increased awareness and created a sense of urgency. PCPs were then asked whether ongoing combination aspirin and warfarin treatments were clinically indicated. If not, the anticoagulation clinic-based aspirin deimplementation team assisted with the discontinuation of aspirin. 33 The intervention was associated with a significant decrease in major bleeding events per month with no increase in thrombotic events.

Nudges can be used to further guide ideas in change management. Nudge theory is a concept in behavioral economics that describes easily avoidable changes to the environment that can influence our behaviors without restricting choice. Increasing evidence suggests that the use of nudges improves clinical workflow and patient outcomes. 34 35 Integrating nudges into the electronic medical record (EMR) can have a significant impact. A proposed example of nudging in anticoagulation care is using the EMR to precalculate the Khorana venous thromboembolism risk score among nonhospitalized patients with cancer and provide an opt-out nudge for thromboprophylaxis in those with a high score. 36 The opt-out nudge options imply preselecting the desirable alternative from the population health perspective as default yet allowing the practitioner to change the choice. To improve the adoption of personalized risk-based thromboprophylaxis of hospitalized surgical patients using the Caprini risk score, 37 automated risk scoring triggered best practice alerts (BPAs) suggesting prophylaxis duration. 38

### Health Systems Engineering (Including Lean Thinking and Six Sigma)

The lean process, initially introduced by the Japanese automaker Toyota in 1940s, aims to improve efficiencies and reduce wasteful practices—briefly, less is better. The Six Sigma framework was established in the 1980s by the American electronics manufacturer Motorola. which aimed to reduce defects in the production process. The name is attributed to the aspirational target that defects should only occur at the 6th standard deviation (sigma), at 3.4 defects per million opportunities. While we are significantly behind this goal in many areas of health care, achieving this target would mean that, using worldwide health care numbers, only 20 chronically anticoagulated patients with atrial fibrillation would receive the wrong perioperative anticoagulation management recommendation each year. 10 Examples of problems relevant to perioperative management which lean systems may look for reduction and simplification include coexistence of multiple triggers to start perioperative anticoagulation instructions when the patient, surgeon, and PCPs call for instructions to the same episode. Optimization of nonutilized talent, as when a minimal risk procedure, which does not need anticoagulation interruption, escalates to a physician appointment when the query may be solved by a nurse or advanced practitioner familiar with the protocol. Waste of transportation time may be eliminated by using a telehealth visit as a preferred method to explain the interruption plan for some high-risk procedures.

Two examples of Lean Thinking come from the United States Veterans Affairs system and health system in Michigan. To reduce waste in staff work and support front-line clinicians with anticoagulation prescribing decisions, the VA implemented a nationwide population management dashboard system to support pharmacist review of prescribed DOACs. The stand-alone dashboard summarizes dose, renal function, age, weight, drug interactions, and refill needs. 39 The dashboard allows pharmacists to focus on patients who need corrective action on anticoagulation management and not spend time reviewing charts for patients who are known to have correct DOAC prescriptions. Another example of stewardship is the use of BPAs generated by the integration of EMRs to facilitate anticoagulation decision-making. In a study by Han et al, BPAs were activated to recommend a referral to an anticoagulation clinic for patients who needed gastrointestinal endoscopy. Patients referred to the anticoagulation clinic had better guideline-adherent medication management. 40 Moreover, this type of intervention also improves patient and physician satisfaction. BPAs can also reduce waste by reducing cancelations of planned procedures due to medication mismanagement. 41

These problem-solving examples illustrate the Define, Measure, Analyze, Improve, and Control process, which is a problem-solving tool used in Six Sigma that is also used in antimicrobial stewardship and other quality improvement projects. 42

In the vignette, the patient is asked to seek authorization and management plan for anticoagulation interruption. This practice puts the burden of care on the patient and generates an asynchronous and confusing loop of opinions among providers. In a lean system, booking the patient to operating room time would send an EMR-generated summary of the bleeding and thrombotic risk factors for this patient to a care navigator. This information would condense a sharable plan of care consistent with defined algorithms for perioperative anticoagulation management that had been previously vetted by a multidisciplinary committee. An opt-out alternative would then be sent to the physician to finalize the order.

## Potential Economic Impact

Consistent and high-quality delivery of care in anticoagulation is only a segment of value care. To standardize the quality delivery to a cost denominator, anticoagulation stewardship units must also study the economic impact of the initiative. In the United States, almost one of every five gross domestic product dollars will be spent on health care. Yet, this cost is disproportionally wasteful and of heterogeneous care value. 43 44 Estimates suggest that approximately one-third of costs are inappropriate, and there is as much as a 20-fold cost variability in health care delivery by site. 18 Perioperative anticoagulation management serves as a good example of health care variability, with the potential that standardized management will improve patient-important outcomes, enable more efficient use of resources and will, in turn, reduce costs. There is a pervasive disconnect between health care protocols and transparent reporting of impact patient outcomes and value care. 45 To this end, evaluation of time-driven, activity-based costing (TDABC), allows comparison of resource allocations to achieve patient outcomes. This method allows the calculation of cost for the entire cycle of care as it calculates cost relative to the specific amount of time a patient uses a resource. TDABC has been used to discuss the utility of management options in severe postthrombotic syndrome and in acute pulmonary embolism management, but wider utilization is due. 46 47 TDABC can help inform choices to adjust workflow utilization in perioperative anticoagulation. To enhance unity in quality delivery, certification in anticoagulation competency should be incentivized.

## Additional Issues in Implementation Perioperative Anticoagulation

The team in charge of implementing perioperative anticoagulation guidelines needs to realize that lack of guideline knowledge is not the only barrier to their use. Meaningful use of the EMR is still low, 18 and manual collection of instructions and patient-level data adds cost. The implementation team needs institutional support to access information technology resources. Accessing these resources is critical both for data collection and the implementation of various strategies. Moreover, these resources are complementary to educational efforts.

In addition, outcome measurement, the main tool for innovation in health care, 48 is not well defined for perioperative anticoagulation, and there is limited integration of patient perspective. While most publications on perioperative anticoagulation address the risk of bleeding and thrombosis, less attention is given to patient-reported perspectives on communication strategies or the integration of virtual visits to facilitate care. It is unclear how best to communicate medication management directly to patients and their caregivers in the perioperative period, especially when uncertainty exists about the postoperative course and appropriate medication management. Moreover, patients are frequently asked to initiate contact with an anticoagulation prescriber armed with less than the complete information needed to determine safe anticoagulation management.

## Conclusion

There is a lag between the identification of new evidence and its adoption in the medical community. Promoting anticoagulation stewardship support at an institutional level can empower both administrators and clinicians to coordinate and facilitate evidence-based changes in practice. A unified approach to perioperative anticoagulation management demands interacting with multiple subspecialties and addressing system barriers. Anticoagulation stewardship units are uniquely poised to guide the implementation process.
